# Supplementary material for: Unveiling Acinetobacter endophylla sp. nov.: A Specialist Endophyte from Peganum harmala with Distinct Genomic and Metabolic Traits
Source: Microorganisms. 2025 Dec 15;13(12):2843. doi: 10.3390/microorganisms13122843 (PMC12735639; doi:10.3390/microorganisms13122843)
Supplement: Supplementary file 1 [file microorganisms-13-02843-s001.zip › microorganisms-4021259-supplementary.pdf]

Supplementary Information

Table S1. Endophytic traits of bacterial strains AGC35 and AGC59 isolated from the foliar endosphere of *Peganum harmala*.

Genes	Proteins	***Acinetobacter endophylla***	***Acinetobacter pitti***
Detoxification	2-dehydropantoate 2-reductase	☗	☗
	glutathione S-transferase	☗	☖
	S-(hydroxymethyl)glutathione dehydrogenase / alcohol dehydrogenase	☗	☗
Plant polymerdegradation/ modification	alpha,alpha-trehalase	☗	☗
	cupin 2 domain-containing protein	☗	☗
Redox potential maintenance	3-hydroxyisobutyrate dehydrogenase	☗	☗
	acetoacetyl-CoA reductase	☗	☗
	aldehyde dehydrogenase	☗	☗
	malate dehydrogenase	☗	☗
Secretion and delivery system	MFS transporter, DHA1 family, multidrug/chloramphenicol efflux transport protein	☗	☗
	type VI secretion system lysozyme-related protein	☗	☖
	type VI secretion system protein	☗	☗
Transcriptional regulator	AraC/DeoR/Lrp/AsnC/LysR families of transcriptional regulators, or	☗	☗
Transporter	ABC transport system ATP-binding/permease protein	☗	☗
	branched-chain amino acid transport system ATP-binding protein	☗	☗
	gluconate 2-dehydrogenase alpha chain	☗	☗
	gluconate 2-dehydrogenase gamma chain	☗	☖
	L-lysine exporter family protein LysE/ArgO	☗	☗
	major facilitator superfamily domain-containing protein 12	☗	☗
	major facilitator superfamily domain-containing protein 2B	☗	☖
	MFS transporter, MHS family, metabolite:H+ symporter	☗	☗
	NAD(P) transhydrogenase	☗	☗

Heavy metal bioremediation	Cobalt-zinc-cadmium resistance protein / Nickel and cobalt resistance protein / Arsenate reductase / Copper resistance protein	☗	☗
Hydrolytic enzymes	Phospholipase D / α-Amylase / Coccaine esterase / Proteases	☗	☗
Indole acetic acid biosynthesis	Indole-3-glycerol phosphate synthase / Tryptophan synthase	☗	☗
Oxidative stress and abiotic stress tolerance	Glutathione S-transferase / Hydroxyacylglutathione hydrolase / Glutathione synthetase / Superoxide dismutase / Polyphenol oxidase / Catalases / Trehalose-6-phosphate synthase, phosphatase / Betaine aldehyde dehydrogenase / Proline/betaine transporter	☗	☗
Phosphorus availability	Pyrroloquinoline quinone biosynthesis / Alkaline phosphatase D / Polyphosphate kinase/ Exopolyphosphatase	☗	☗
Salicylic acid biosynthesis	Isochorismate synthase / Chorismate synthase / Acetyl-coenzyme A carboxylase carboxyl transferase	☗	☗
Sulfur availability and transport	Alkanesulfonate monooxygenase / Methanesulfonate monooxygenase / NADPH-dependent flavin mononucleotide reductase ArsH	☗	☗
Temperature adaptation	Cold shock protein / Small heat shock protein / Heat shock protein / Heat-inducible transcription repressor	☗	☗
Other	2-isopropylmalate synthase	☗	☗
	diaminopimelate decarboxylase	☗	☗

☗ presence

☖ absence

Table S2. Main characteristics of the draft genome assemblies of endophytic bacteria isolated from *Peganum harmala*.

Plant Origin	Bacterial Species Names	Strain ID	Assembler	Largest Contig	# Contigs	N50	Genome Length	GC (%)	Completeness (%)	Contamination (%)	Depth (X)	Protein sequence (	tRNA aa	16S rRNA (%)	Accession number (SR.
Ph-F	*Acinetobacter endophylla*	AGC35	MaSuRCA	196,371	329	72,460	3,749,590	42.58	99.93	0.72	73	4,068	20	97.8	SRR29855794
Ph-F	*Acinetobacter pitti*	AGC59	MaSuRCA	577,240	216	260,442	3,899,124	38.73	98.85	0.57	104	3,845	20	96.1	SRR29855731

Table S3. Taxonomic identification of endophytic bacterial isolates from *P. harmala* based on 16S rRNA gene sequencing using the Sanger method.

Bacterial Species Names	Strain ID	Sequence lenght	Identity (%)	Coverge (%)	Closest species name	Accession number
Acinetobacter endophylla	**AGC35**	**861**	**98.4**	**100**	***Acinetobacter lwoffii***	***PV739381***
Acinetobacter pitti	**AGC59**	**847**	**98**	**100**	***Acinetobacter pittii***	***PV739392***

Table S4. Biolog phenotypic profiling results for strain AGC35.

Group	Characteristic	Reults
Disaccharides & Trisaccharides	Dextrin	(-)
	D-Maltose	(-)
	D-Trehalose	(-)
	D-Cellobiose	(-)
	Gentiobiose	(-)
	Sucrose	(-)
	D-Turanose	(-)
	Stachyose	(-)
	D-Raffinose	(-)
	a-D-Lactose	(-)
	D-Melibiose	(-)
Monosaccharides & Derivatives	a-D-Glucose	(-)
	D-Mannose	(-)
	D-Fructose	(-)
	D-Galactose	(-)
	3-Methyl Glucose	(+/-)
	D-Fucose	(+)
	L-Fucose	(+)
	L-Rhamnose	(+)
	N-Acetyl-D-Glucosamine	(-)
	N-Acetyl-b-D-Mannosamine	(-)
	N-Acetyl-D-Galactosamine	(-)
	N-Acetyl Neuraminic Acid	(-)
	b-Methyl-D-Glucoside	(-)
	D-Salicin	(-)
Sugar Phosphates	D-Glucose-6-PO4	(+)
	D-Fructose-6-PO4	(-)
Sugar Acids	D-Galacturonic Acid	(-)
	L-Galactonic Acid Lactone	(+)
	D-Gluconic Acid	(-)
	D-Glucuronic Acid	(-)
	Glucuronamide	(-)
	Mucic Acid	(-)
	Quinic Acid	(-)
	D-Saccharic Acid	(-)
Amino Acids	L-Alanine	(+)
	L-Arginine	(-)
	L-Aspartic Acid	(-)
	D-Aspartic Acid	(-)
	L-Glutamic Acid	(+)
	L-Histidine	(-)
	L-Pyroglutamic Acid	(-)
	L-Serine	(-)
	D-Serine	(+)
	g-Amino-Butyric Acid	(-)
Peptides	Glycyl-L-Proline	(-)
Organic Acids	Acetic Acid	(+/-)
	Formic Acid	(-)
	Propionic Acid	(-)
	Acetoacetic Acid	(-)
	Citric Acid	(-)
	D-Malic Acid	(-)
	L-Malic Acid	(-)
	Bromo-Succinic Acid	(+/-)
	a-Keto-Glutaric Acid	(-)
	p-Hydroxy-Phenylacetic Acid	(-)
	a-Hydroxy-Butyric Acid	(-)
	β-Hydroxy-D,L-Butyric Acid	(+/-)
	a-Keto-Butyric Acid	(-)
Esters	Methyl Pyruvate	(-)
	D-Lactic Acid Methyl Ester	(-)
	L-Lactic Acid	(+)
Alcohols and Polyols	D-Sorbitol	(-)
	D-Mannitol	(+)
	D-Arabitol	(-)
	myo-Inositol	(-)
	Glycerol	(-)
Polymers and Other Carbon Sources	Gelatin	(-)
	Pectin	(-)
	Tween 40	(-)
Nucleotides / Nucleosides	Inosine	(-)
Antibiotics and Inhibitors	Aztreonam	(-)
	Fusidic Acid	(-)
	Guanidine HCl	(-)
	Lincomycin	(-)
	Minocycline	(-)
	Nalidixic Acid	(-)
	Rifamycin SV	(-)
	Troleandomycin	(-)
	Vancomycin	(-)
pH Indicators	pH 5	(-)
	pH 6	(-)
Osmotic Stress (NaCl)	1% NaCl	(-)
	4% NaCl	(-)
	8% NaCl	(-)
Ionic Stress	1% Sodium Lactate	(-)
	Lithium Chloride	(-)
	Sodium Butyrate	(+/-)
	Sodium Bromate	(+/-)
Other Reagents	Niaproof 4	(+)
	Tetrazolium Violet	(-)
	Tetrazolium Blue	(-)

(+) Presene, (−) Abscence, (+/−) Borderline

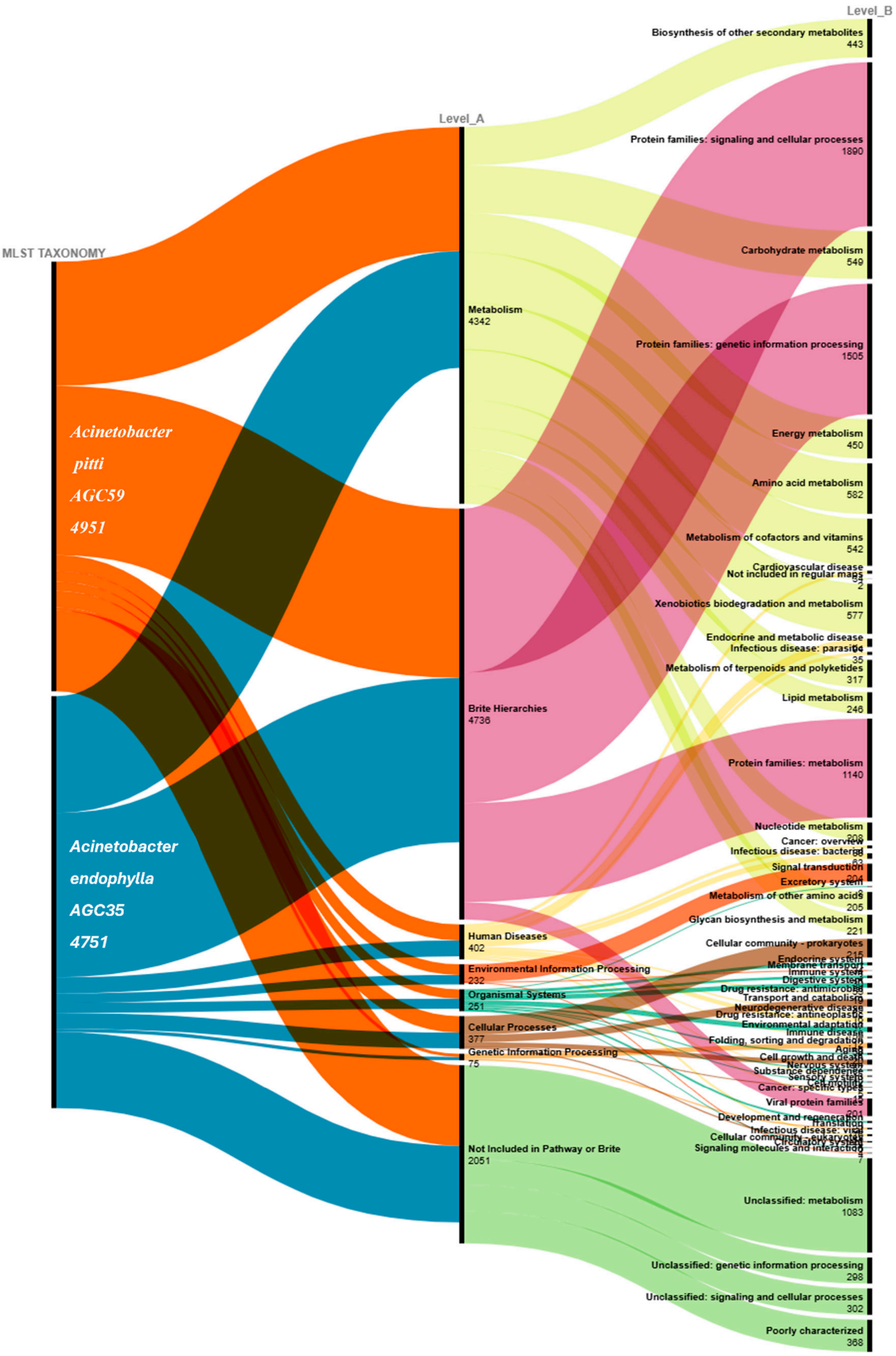

Figure S1. KEGG functional categorization: Sankey diagram showing the distribution of K-numbers across BRITE hierarchies, illustrating genes associated with metabolic pathways, genetic

information processing, and cellular processes for strains AGC35 and AGC59 isolated from the foliar endosphere of *Peganum harmala*.
